# Relationships between variance in electroencephalography relative power and developmental status in infants with typical development and at risk for developmental disability: An observational study

**DOI:** 10.12688/gatesopenres.12868.2

**Published:** 2018-11-15

**Authors:** Andrew Hooyman, David Kayekjian, Ran Xiao, Crystal Jiang, Douglas L. Vanderbilt, Beth A. Smith

**Affiliations:** 1 Motor Behavior and Neurorehabilitation Laboratory, Division of Biokinesiology and Physical Therapy, University of Southern California, Los Angeles, CA, 90089, USA; 2 Infant Neuromotor Control Laboratory, Division of Biokinesiology and Physical Therapy, University of Southern California, Los Angeles, CA, 90089, USA; 3 Department of Physiological Nursing, School of Nursing, University of California San Francisco, San Francisco, CA, 94143, USA; 4 Department of Pediatrics, Division of General Pediatrics, Keck School of Medicine, University of Southern California, Los Angeles, CA, 90089, USA

**Keywords:** infant development, electroencephalography, movement system, motor skills

## Abstract

**Background:** Electroencephalography (EEG) is a non-invasive tool that has the potential to identify and quantify atypical brain development. We introduce a new measure here, variance of relative power of resting-state EEG. We sought to assess whether variance of relative power of resting-state EEG could predict i) classification of infants as typical development (TD) or at risk (AR) for developmental disability, and ii) Bayley developmental scores at the same visit or future visits.

**Methods:** A total of 22 infants with TD participated, aged between 38 and 203 days. In addition, 11 infants broadly at risk participated (6 high-risk pre-term, 4 low-risk pre-term, 1 high-risk full-term), aged between 40 and 225 days of age (adjusted for prematurity). We used EEG to measure resting-state brain function across months. We calculated variance of relative power as the standard deviation of the relative power across each of the 32 EEG electrodes. The Bayley Scales of Infant Development (3 ^rd^ edition) was used to measure developmental level. Infants were measured 1-6 times each, with 1 month between measurements.

**Results:** Our main findings were: i) variance of relative power of resting state EEG can predict classification of infants as TD or AR, and ii) variance of relative power of resting state EEG can predict Bayley developmental scores at the same visit (Bayley raw fine motor, Bayley raw cognitive, Bayley total raw score, Bayley motor composite score) and at a future visit (Bayley raw fine motor).

**Conclusions:** This was a preliminary, exploratory, small study. Our results support variance of relative power of resting state EEG as an area of interest for future study as a biomarker of neurodevelopmental status and as a potential outcome measure for early intervention.

## Introduction

Early detection of atypical neurological development increases the potential for successful intervention, as a body of basic science laboratory data supports that a wide variety of interventions, from environmental enrichment to hypothermia or implantation of stem cells, can enhance cerebral plasticity during development ^1^. Emerging data also support that clinical interventions can increase the developmental potential of children, rather than presuming a predetermined potential ^1^. Accordingly, early therapy intervention should have the greatest benefit on neural development and functional outcomes. However, there is a crucial roadblock here. In order to help guide and monitor interventions seeking to promote healthy brain development in the early years, we need suitable measures of fetal and infant brain function and development ^2^ prior to functional impairments emerging.

Electroencephalography (EEG) offers one non-invasive tool with the potential to identify and quantify atypical brain development. While EEG has been used since the early 1900s to diagnose conditions such as sleep and chronic seizure disorders, it has more recently been investigated as a screening tool in the neonatal intensive care unit for high-risk infant populations ^3^. The rapidly growing field of infant EEG seeks to uncover specific abnormalities in activity patterns or key features, and whether these are predictive of short-term and long-term risks or outcomes ^3^.

Previous research has determined that EEG measures have some capacity in infancy to predict later functional outcomes. El-Dib and colleagues ^4^ demonstrated the ability of an EEG measure of continuity, minimum amplitude, bandwidth, and cycling within the first week of life to predict poor outcome (death or severe delay on Bayley Scales of Infant Development, version 2) at 4 months corrected age in 55 infants born pre-term (26–29 weeks gestational age) or with very low birth weight (less than 1500 g). For poor outcomes, EEG had a sensitivity of ~30%, specificity of ~90%, positive predictive value of ~60% and negative predictive value of ~80% ^4^. They did not use cross validation to confirm accuracy of model. Hayashi-Kurabashi *et al*. ^5^ demonstrated relationships between background activity of EEG within the 36 days of life and a diagnosis of developmental delay or cerebral palsy at 12–18 months corrected age in 333 infants born pre-term (less than 36 weeks gestational age). For prediction of a later diagnosis, EEG had a sensitivity of 50–61%, specificity of 74–86%, positive predictive value of 27–38% and negative predictive value of 91–93% ^5^. They did not use cross validation to confirm accuracy of the model. An additional study by Périvier and colleagues ^6^ also related clinical EEG data to infant outcomes. They found that out of 1744 preterm infants (less than 32 weeks gestational age), 422 had non-optimal outcomes at 2 years. A clinical rating scale that considered multiple aspects of abnormality of the EEGs performed in early infancy (up to 33 weeks post-menstrual age) had good specificity (0.95) but low sensitivity (0.16) for predicting non-optimal outcomes. Non-optimal outcomes were non-optimal neuromotor function or abnormal psychomotor development across any of a number of clinical measures ^6^. Although EEG measures show some promise, to date they have only provided a piece of the puzzle. In a number of studies where outcomes were predicted using EEG it has been recommended that EEG assessment be combined with other clinical measures ^4, 6, 7^. More effort is needed to determine the salient factors of EEG to be included for an optimally accurate and efficient prediction of neurodevelopmental outcomes, which led us to explore a new measure here.

 We introduce a new measure here, variance of relative power of resting state EEG. We calculated variance of relative power as the standard deviation of the relative power across each of the 32 EEG electrodes. We postulate that higher variance may represent less organized cortical activity and be an intuitive and useful metric for identifying and quantifying atypical brain development within the first months of life. As such, higher variance may represent a salient factor of EEG to include for an optimally accurate and efficient prediction of neurodevelopmental outcomes.

## Methods

### Recruitment

This was a preliminary study to explore potential relationships of interest between EEG and developmental status, and we used a sample of convenience. Data were collected between 17 February 2015 and 18 June 2016. A total of 22 infants with typical development (TD) participated, between 38 and 203 days of age ( Table 1). There were 2 infants with TD measured once, with the other 20 infants measured once per month for 3 to 6 visits. A total of 11 infants broadly at risk (AR) for developmental disability participated (6 high-risk pre-term, 4 low-risk pre-term, 1 high-risk full-term), aged between 40 and 225 days of age (adjusted for prematurity; Table 1). Infants AR were assessed once per month for 3 to 5 visits. Assessments started as close to 1 month of age as possible, and continued until the infant successfully reached and grasped a toy with high skill. Inclusion criteria (TD): infants were from singleton, full-term births (over 38 weeks). Exclusion criteria (TD): infants experiencing complications during birth, or with any known visual, orthopedic or neurologic impairment at the time of assessment, or with a score at or below the 5 ^th^ percentile for their age on the Bayley Scales of Infant Development, 3 ^rd^ edition ^8^ would have been excluded at the time of testing. Inclusion criteria (AR): AR infants were born before 36 weeks of gestation (low risk) or defined as at high risk for developmental delay per the definition of the state of California ^9^. Infants AR were a broad group and not homogenous to one risk profile or level for this preliminary study. Exclusion criteria (AR): infants with unstable medical conditions would have been excluded. Infants were recruited by a member of the research team in-person at the Eisner Health Clinic (Los Angeles, CA, USA) and Children’s Hospital of Los Angeles (Los Angeles, CA, USA). Infants were also recruited by referral from Ventura County Medical Center (Ventura, CA, USA), through fliers distributed or posted at the University of Southern California (USC), and by word of mouth. This study was approved by the Institutional Review Board of the USC (HS-14-00690). A parent or legal guardian signed an informed consent form prior to their infants’ participation.

**Table 1.** Infant characteristics.

Visit	Group	Descriptive statistic	Age (in days, adjusted for prematurity as applicable)	Weight (kg)	Body length (cm)	Head circumference (cm)
1	At risk, full term	N	1	1	1	1
		Mean	168	6.3	64.5	39.7
		Std. Deviation				
		Minimum	168	6.3	64.5	39.7
		Maximum	168	6.3	64.5	39.7
	Preterm, high risk	N	6	6	6	6
		Mean	91	5.5	59.6	38.9
		Std. Deviation	35	0.9	4.6	1.7
		Minimum	68	4.5	53.5	37.5
		Maximum	162	7.1	67.5	42.0
	Preterm, low risk	N	4	4	4	4
		Mean	73	5.8	60.5	37.8
		Std. Deviation	38	0.8	4.9	2.1
		Minimum	40	4.9	56.3	35.0
		Maximum	127	6.8	67.5	40.0
	Typical development	N	22	22	22	22
		Mean	105	6.7	62.6	40.1
		Std. Deviation	44	1.0	3.5	1.9
		Minimum	38	5.0	57.5	37.5
		Maximum	203	8.9	69.5	43.6
3	At risk, full term	N	1	1	1	1
		Mean	230	7.6	68.0	40.2
		Std. Deviation				
		Minimum	230	7.6	68.0	40.2
		Maximum	230	7.6	68.0	40.2
	Preterm, high risk	N	6	6	6	6
		Mean	156	6.7	63.6	41.2
		Std. Deviation	35	0.8	3.8	1.5
		Minimum	132	6.0	59.0	40.0
		Maximum	225	8.1	70.0	44.0
	Preterm, low risk	N	4	4	4	4
		Mean	137	7.2	63.1	40.6
		Std. Deviation	37	0.7	1.9	1.1
		Minimum	103	6.3	61.0	39.0
		Maximum	190	7.8	65.0	41.2
	Typical development	N	20	20	20	20
		Mean	158	7.7	66.9	41.5
		Std. Deviation	34	0.9	3.8	1.6
		Minimum	97	6.2	56.0	38.0
		Maximum	203	9.1	71.5	45.0

### Assessment

Infants were measured primarily in the family’s home, in the morning. Per the family’s preference, three families came to the laboratory at the USC Health Science Campus for some of their visits. Each visit lasted for around 1 hour. At each visit, the infant’s weight, body and limb lengths, and head and limb circumferences were measured. Motor, cognitive and language development were assessed using the Bayley Scales of Infant Development, 3 ^rd^ edition ^8^. A small wearable sensor was placed on each arm, and 5 minutes of video of the infant’s spontaneous movement in supine was recorded. Wearable sensors remained on for the rest of the day. Wearable sensor data are reported in previous publications ^10, 11^ and are not discussed further here. The parent or guardians’ highest level of education completed was recorded. Families were compensated for each visit. Data were stored on a password-protected server or in a REDCap electronic database (version 6.14.2) hosted by USC.

### Electroencephalography assessment

During each visit, EEG data were acquired using a Biosemi system with 32-electrode infant headcaps (standard 10/20 system) at sampling rate of 512 Hz. Infants sat on the lap of a caregiver. First, 2 trials of 20-second resting-state EEG data were recorded. During resting state recording, a lighted, spinning globe toy was presented out of participants’ reach to attract their visual attention and minimize head and body movement. This is standard in infant EEG data collection ^6, 7^. Next, arm reaching skill was assessed using 20-second blocks where a toy was presented at midline within reaching distance of the infant alternating with 20-second blocks without a toy to reach for. This was repeated five times. Finally, another session of resting-state EEG data were collected, similar to the first session.

### Data analyses

***EEG analyses*.** EEG analysis methods are described in detail in a previous publication ^12^. Only resting state EEG data were analyzed here, ranging from 14–82 seconds. Resting-state EEG variables explored here are individual power, relative power, and variance of relative power. Briefly, EEG data from all electrodes were re-referenced to the average of T7 and T8. Next, a bandpass infinite impulse response filter (0.3–30 Hz) was applied to the re-referenced data. Resting EEG segments were epoched and noisy segments were rejected. After rejection, remaining EEG data from 11 infants AR and 22 infants with TD were: AR visit 1 = 11, AR visit 3 = 9, TD visit 1 = 21, TD visit 3 = 13. Power spectral density (PSD) was estimated on these preprocessed EEG data using the “pwelch” function in MATLAB (ver. 2016A, MathWorks Inc., Natick, MA, USA). PSDs were transformed into relative powers so that spectral activities from all individual sessions were directly comparable. The relative powers were calculated between 0 and 30 Hz. For each frequency bin within this range and each electrode, relative power was computed by dividing PSD by the sum PSD from all bins. Variance of relative power was calculated as the standard deviation of the 32 relative power measurements for each infant, calculated by taking the standard deviation of peak power across each channel.

***Bayley scales of infant development*.** Bayley scales of infant development version 3 raw scores for gross motor, fine motor, expressive language, receptive language, and cognition were transformed into composite scores and percentile ranks by age corrected for gestational age less than 38 weeks for motor, cognitive and language domains. Bayley composite scores are determined in 2-week, age-normalized windows and created to have a range of 40–160, mean of 100 and SD of 15. Composite score classification are: 130 and above, very superior; 120–129, superior; 110–119, high average; 90–109, average; 80–89, low average; 70–79, borderline; 69 and below, extremely low ^8^. An infant developing at a steady rate would be expected to have composite scores that remained steady over time.

***Statistical analyses*.** Logistic regression was conducted to predict at-risk status of infants in the cohort using resting state EEG data recorded at visit 1. Leave-one-out cross-validation was performed as a method to confirm accuracy of logistic regression model. Multivariate linear regression was conducted to predict current (visit 1) and future (visit 3) Bayley scores using resting-state EEG data. Statistical analyses were performed using R, version 3.5.1. Bayley score models were compared using analysis of variance. It is important to note that the EEG analysis (RX) and the statistical analysis (AH) were performed independently from one another.

### Prediction of AR status

The resting state data for each infant was derived into individual power and relative power readings from each electrode, 32 electrodes in all. Raw data are available on figshare ^13^. Initially, all 32 power and relative measurements from visit 1 were input into various machine learning algorithms (including K-nearest Neighbor, Support Vector Machine, and Logistic Regression with L1 regularization) to predict the infant’s at-risk status. Leave-one-out cross-validation was performed on each model. Then, the variance of relative powers across 32 electrodes were computed as input features for logistic regression to test their predictive efficacy for the classification task.

### Prediction of same visit (1st visit) Bayley scores

Multivariate linear regression was conducted to predict current and future Bayley scores to identify if variance of relative power made a significant contribution to prediction. We designed 12 different linear regression models with each one specific to a different category/composite of Bayley score ( Table 2). First, we implemented models that only used age in days to predict each Bayley category. These models did not use variance of relative power as a predictor and thus served as the baseline models to be compared against the baseline models plus variance of relative power.

**Table 2.** Between-model statistical results.

Clinical test	Adj R^2 null	Adj R^2 full	BIC null	BIC full	ANOVA *p*-value
Bayley raw fine motor	0.59	0.64	159.89	157.89	0.03
Bayley raw gross motor	0.61	0.63	169.19	169.99	0.12
Bayley raw receptive language	0.11	0.13	111.22	112.99	0.22
Bayley raw expressive language	0.28	0.27	107.43	110.20	0.43
Bayley raw cognitive	0.65	0.71	164.44	161.42	0.02
Bayley total raw score	0.69	0.74	232.00	228.62	0.01
Cognitive composite	0.17	0.22	264.50	264.83	0.1
Cognitive percentile rank	0.06	0.07	277.45	279.45	0.25
Language composite	0.11	0.11	255.08	257.46	0.32
Language percentile composite	0.00	0.03	202.36	203.60	0.18
Motor composite	0.19	0.30	267.77	265.09	0.02
Motor percentile rank	0.09	0.17	308.41	307.94	0.06

Each model was examined for assumptions of linear regression (i.e. heteroschedasticity and multicollinearity). Visual inspection of residuals and analysis of correlation between predictors revealed that each model maintained their regression assumptions. A baseline statistical model (a model that only included age in days and at-risk status) was compared to a nested model of the baseline model features plus variance of relative power to determine significant predictive effects of variance of relative power beyond baseline prediction. We used analysis of variance to determine significant predictive effects of variance of relative power across Bayley scores.

### Prediction of future visit (3 ^rd^ Visit) Bayley scores

A multivariate linear regression was conducted with age, at-risk status, and variance of relative power at visit 1 to predict Bayley scores at visit 3. On average, visit 3 took place 60 days after visit 1. The 3-regressor model using age, at-risk status, and variance of relative power was compared against a 2-regressor model using age and at-risk status only.

## Results

### Prediction of AR status

**Figure 1.**
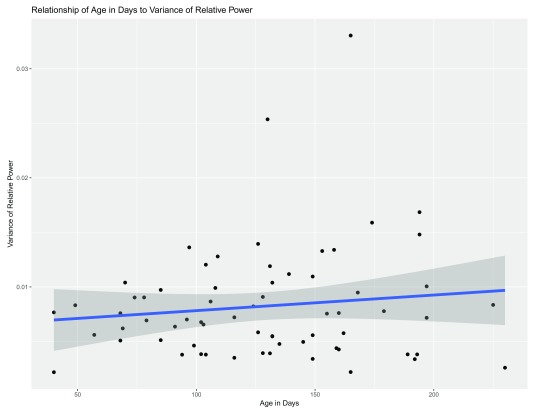
Age in days does not predict overall variance of relative power. Blue line represents linear regression fit, shaded area is standard error of fit.

Leave-one-out cross-validation was performed on each machine learning model to predict at-risk status among 32 infants (11 at-risk) with a mean age of 90 days. Only modest accuracy was identified with typically a high false negative rate for features from conventional metrics (i.e., power and relative power). On the other hand, variance of relative power was calculated as standard deviation of the 32 relative power measurements for each infant and was used as the only predictor within the model. A test of the full model (at-risk status ~ variance of relative power) compared to a baseline model (at-risk status ~ intercept only) was statistically significant, indicating that variance of relative power accurately classified at-risk status (chi square = 7.64, *p* < 0.01, *df* = 2, odds ratio = 1.18). Conversely, at-risk status significantly predicted variance of relative power ( *p* < 0.01, *F* = 8.33, R ^2^ = 0.217, *df* = 2). A designation of at-risk was associated with higher variance of relative power. Interestingly, as shown in Figure 1, age in days did not predict variance of relative power ( *p* > 0.05).

**Figure 2.**
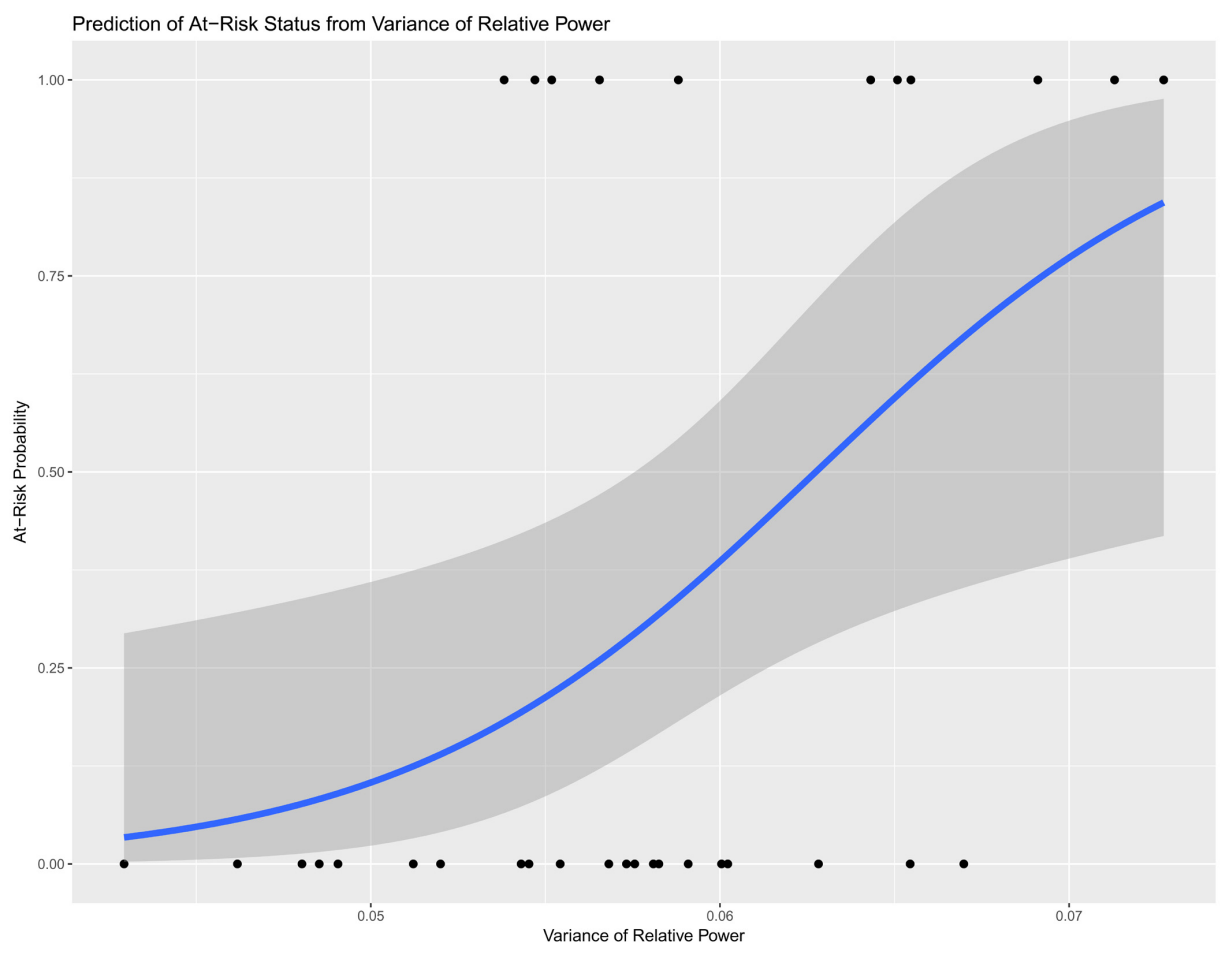
Infants with higher variance of relative power across all 32 electroencephalography electrodes had a higher probability of being classified as at-risk. Blue line represents logistic regression fit, shaded area is standard error of fit. Superior data points are variance of relative power measures for infants at risk, inferior data points are variance of relative power measures for infants with typical development.

Leave-one-out cross-validation was performed using the identified logistic model to create a confusion matrix. Results demonstrated an overall accuracy of 75%, with a true negative rate of 86% (18/21) and a true positive rate of 55% (6/11). Results of the analysis demonstrated that an infant with higher variance of relative power across all EEG electrodes had a higher probability of being classified as AR ( Figure 2).

### Prediction of same visit (1st visit) Bayley scores

Results demonstrated that variance of relative power provided a significant contribution to 1st visit scores of Bayley raw fine motor, Bayley raw cognitive, Bayley total raw score, and motor composite score ( *p* < 0.05, see Table 2).

### Prediction of future visit (3 ^rd^ Visit) Bayley scores

The 2-regressor model was significantly different from a baseline model ( *p* < 0.001, *F* = 15.61, adjR ^2^ = 0.58, *df* = 2). Analysis of variance was used to compare the 2-regressor model to the 3-regressor model at alpha = 0.05. This result demonstrated that the addition of variance of relative power from visit 1 contributed to prediction of Bayley raw fine motor score at visit 3 ( *p* < 0.001, *F* = 14.13, adjR ^2^ = 0.65, *df* = 3). Overall, variance of relative power was able to contribute an extra 7% of variance explained compared to a 2-regressor model using measures of age and at-risk status.

## Discussion

Our main findings were: i) variance of relative power of resting state EEG can predict classification of infants as TD or AR, and ii) variance of relative power of resting state EEG can predict Bayley developmental scores at the same visit (Bayley raw fine motor, Bayley raw cognitive, Bayley total raw score, Bayley motor composite score) and at a future visit (Bayley raw fine motor).

### Prediction of AR status

Higher variance of relative power predicted AR status, while age in days did not. We propose that higher variance may represent less organized cortical activity associated with an atypical trajectory of brain development. This is consistent with the use of ‘EEG complexity’ as a measure to distinguish infants with TD from infants at high risk for autism spectrum disorders ^14^. While age must certainly be considered—as a bias toward synaptic formation leads to a peak in synaptic density between 6–18 months of age, followed by a shift to synaptic pruning ^15^—these studies imply that trajectories between populations of infants are diverging along the course of development. It is important to note that these studies both include infants who are at risk, without considering their ultimate outcomes (diagnoses). Further, we included both low- and high-risk infants in this study. We did not expect the AR infants to be a homogenous group with regards to their brain development and EEG data, rather we expected the AR infants to be different than the TD group, potentially in different ways across infants. Predicting or classifying risk status is not interchangeable with predicting future developmental outcomes/diagnoses.

### Prediction of Bayley scores

Our results showed that variance of relative power provides a significant contribution to 1st visit (same visit) score prediction of Bayley raw fine motor, Bayley raw cognitive, Bayley total raw score, Bayley motor composite score. Further, we found that variance of relative power from visit 1 contributes to prediction of Bayley raw fine motor score at visit 3. This is consistent with our previous work, where we found a relationship between a different measure, EEG coherence, and Bayley raw fine motor and gross motor scale scores in infants with TD (the same sample of infants with TD as included here) ^12^.

 Previous research in infants with TD has also found relationships between EEG measures of power and coherence and motor and cognitive skill performance in infants. One study found a relationship between power in the alpha band and crawling onset in 5- to 7-month-old infants with TD ^16^. Another study demonstrated differences in the power and coherence of EEG signals of 7- to 12-month-old infants with TD in relation to success with a cognitive skill, the A-not-B task (object permanence). Infants who were successful displayed changes in frontal EEG power and increased anterior-posterior brain region coherence compared to infants who were not successful. The changes in EEG were attributed to increased organization and excitability in the frontal region ^16^. The researchers also demonstrated differences in the power and coherence of EEG signals of 8-month-old typically developing infants with various amounts of crawling experience ^6^ and, recently, in 12-month-old typically developing infants with various amounts of walking experience ^13^.

 Taken together, these studies link brain function, as measured by EEG, to motor and cognitive skill performance across various EEG measures and skills. Our study is unique as the infants here are younger than previous studies, and we have included infants AR in addition to infants with TD. 

### Limitations and future directions

This was a preliminary study in a small sample of infants. Our goal was to highlight potential relationships of interest to be pursued in future, larger, adequately powered studies. In an effort to avoid biased findings based on observations from a small data set, we conducted the EEG analysis and statistical analyses independently. In addition to the small sample size, our study is limited both by factors related to EEG as a tool and by factors related to studying infant development.

 EEG as a tool has known limitations. EEG power is sensitive to non‐neural factors like thickness and shape of tissues between electrodes and the cortex, as well as recording noise due to differences in hair thickness, the fit of the cap, or differing amounts of eye movement between participants. One way we addressed this was by using the relative power instead of the absolute power, another way was by showing that there were no systematic changes with age in overall variance ( Figure 1). It is also important to note that EEG is not a direct measure of cortical activity, so our proposal that higher variance may represent less organized cortical activity may or may not be valid. Future work that directly measures cortical activity is needed.

There are many potential factors that likely influence developmental rate and outcomes in infants with TD and AR, and the same factor may or may not have similar effect strength in each group. Potential contributing factors to examine include: amount and type of movement experience, quality of caregiver–infant interaction, parenting style, cultural expectations, birth order, socioeconomic status, physical growth rate, nutritional status, amount and quality of sleep, personality/motivation, and genetics. Additionally, individual EEG predictors show limited power in predicting outcomes. There is the potential to aggregate these together as features to feed into machine learning algorithms for classification and prediction. We hope to pursue larger, more complex predictive models in future work with a larger sample. Adding EEG measures such as coherence and synchronization of oscillations might increase predictive power, so might including structural brain imaging data or clinic variables. Understanding the relative contribution of each factor to predicting outcomes, as well as their responsiveness to intervention, will be key to providing early intervention to reach optimal developmental potential in infants AR.

This was a preliminary, exploratory, small study of the potential importance of variance of relative power, as measured by resting state EEG data. Our results support variance of relative power as an area of interest for future study as a biomarker of neurodevelopmental status and as an outcome measure for intervention in infants AR. Higher variance may represent less organized cortical activity and be an intuitive and useful metric for identifying and quantifying atypical brain development within the first months of life. We see the potential to combine variance of relative power with other EEG and clinical measures identified in previous studies and to leverage these multiple features using machine learning techniques to improve predictive reliability.

## Conclusions

Infant development is a variable and complex process. As a field, we are starting to determine how and when we can intervene in infants AR to have a positive impact on developmental outcomes. Our findings here, of the ability of variance of relative power of EEG to predict classification of infants as TD or AR and Bayley developmental scores, supports the potential of using variance of relative power of EEG to trace out and classify the developmental trajectories of the nervous system.

## Data availability

A spreadsheet with resting state relative power EEG data and Bayley Scales of Infant Development Scales (version 3) scores for each participant at each assessment is available at figshare: https://doi.org/10.6084/m9.figshare.6994946 ^13^.

Data are available under the terms of the Creative Commons Zero “No rights reserved” data waiver (CC0 1.0 Public domain dedication).
