# Supplementary material for: Relationships between variance in electroencephalography relative power and developmental status in infants with typical development and at risk for developmental disability: An observational study
Source: Gates Open Res. 2018 Nov 15;2:47. Originally published 2018 Sep 11. [Version 2] doi: 10.12688/gatesopenres.12868.2 (PMC6266744; doi:10.12688/gatesopenres.12868.2)
Supplement: Peer review history

# Peer review: Reviewer response for version 2

**DOI:** 10.21956/gatesopenres.13984.r26765

**Published:** 2018-11-22

**Authors:** Wanze Xie

**Affiliations:** 1 Boston Children's Hospital, Harvard Medical School, Boston, MA, USA

The authors have addressed my concerns in their revision.

I have read this submission. I believe that I have an appropriate level of expertise to confirm that it is of an acceptable scientific standard.

# Peer review: Reviewer response for version 1

**DOI:** 10.21956/gatesopenres.13952.r26655

**Published:** 2018-10-01

**Authors:** Caterina Piazza

**Affiliations:** 1 Bioengineering Lab, Scientific Institute, IRCCS Eugenio Medea, Lecco, Italy

The manuscript describes an interesting preliminary study proposing a new EEG measure (i.e. variance of relative power of resting state EEG) as an early biomarker for the prediction of the developmental status. Results seem to suggest the potential of using this measures to evaluate developmental trajectories, since it was able to classify infants with typical development (TD) and at risk(AR) infants and it predicted some Bayley scores both at the same time of the EEG recording and 3 months later.

 The study is clearly and accurately presented. However, some specific comments that should be addressed by the authors are listed below.

1. The variance of relative power was calculated by taking the standard deviation of peak power in the frequency band 0-30Hz. The position of this peak should be reported, I expected that in most cases it was found in the low frequencies. Why did you decide to use this broad band? Did you try to calculate the same measure in the different EEG frequency bands (i.e. theta, alpha beta, etc.).
2. It is not clear if the resting-state EEG measures you took into account (i.e. individual power, relative power and variance of relative power) were computed on all the resting state trails you recorded (i.e. the 2 trails at the beginning of your experiment and the trial at the end of the procedure).

The variance of relative power seem to be a promising biomarker. However I think that your hypothesis that higher variance may represent less organized cortical activity should be better investigated. EEG scalp-based data measure surface potential changes caused by a combination of underlying signals from various sources within the brain, as well as extra-brain sources. In my opinion there are a lot of factors that may potentially contribute to higher variance at the scalp level.

I have read this submission. I believe that I have an appropriate level of expertise to confirm that it is of an acceptable scientific standard.

# Response

**Published:** 2018-11-07

**Authors:** Beth A. Smith

Thank you for your insightful review. In response:

1. We have looked at power variance in the individual frequency bins and peak power is typically within theta. One analysis looking at the alpha frequency was also predictive of 3rd visit scores using 1st visit resting state EEG. We did try prediction within each frequency brand specifically and we found looking across the spectrum to be the most robust.

2. The resting state data came from both periods, to include as much clean data as possible. We have added a sentence in the data analyses section, Only resting state EEG data were analyzed here, ranging from 14-82 seconds”. The first part of the sentence addresses a concern from another Reviewer.

Final comment: We agree! We have been careful to say that higher variance in EEG power may represent less organized cortical activity, as opposed to state that it does represent less organized cortical activity. We have added the possibility of noisier data due to extensive eye movements for some infant participants in the limitations section. We also added a sentence in the limitations section,  “It is also important to note that EEG is not a direct measure of cortical activity, so our proposal that higher variance may represent less organized cortical activity may or may not be valid. Future work that directly measures cortical activity is needed.”

# Peer review: Reviewer response for version 1

**DOI:** 10.21956/gatesopenres.13952.r26662

**Published:** 2018-09-24

**Authors:** Wanze Xie

**Affiliations:** 1 Boston Children's Hospital, Harvard Medical School, Boston, MA, USA

The manuscript describes an EEG-study investigating the prediction of developmental status in infants (TD or AR for developmental disability) and their cognitive outcomes (Bayley scores) using variance in EEG power across electrodes. There were 22 typical developing infants and 11 infants broadly at risk for developmental disability included in the EEG analysis. The variance of EEG relative power across 32 electrodes was calculated using resting-sate EEG data. A logistic regression model was used to predict infants’ AR status, and multivariate linear regressions were conducted to predict the effects of variance in EEG power on infants’ concurrent and future Bayley scores. The authors found that variance of EEG power can classify infants’ developmental status and predict the outcomes/scores of a few Bayley subscales. 

 There is accumulating evidence supporting the capacity of EEG measures to predict functional outcomes in childhood, and thus it is important to seek for efficient and suitable EEG metrics to optimize the prediction. While this work has the potential to advance the field further, there are several issues requiring revision or clarification.They are listed below in the order of appearance in the manuscript.

1. Why does higher variance in EEG power represent less organized cortical activity? This assumption has been made in the introduction and discussion without solid evidence supporting it. Please either cite studies for this assumption or analyzing the network organization to examine the association between variance in EEG power and cortical organization. This is important because other factors can also contribute to higher variance of EEG power across electrodes, such as noisier data in the frontal electrodes due to extensive eye movements for some infant participants.
2. What is the rationale for using variance of relative EEG power to predict developmental status in infants, as well as their cognitive outcomes? Is it because this measure has been tested and validated in the adult literature, or it is just one of the few measures tested by the authors that worked the best?
3. The presentation of the EEG paradigm could be clearer. My understanding is that 2 trials of 20s resting-state EEG data were recorded, followed by a couple of “arm reaching” trials, and then an additional 2 resting-state trials were presented. Is this correct? If so, did the authors use all the data (2 RS + the Arm R trials + 2 RS?) or just the 4 resting-state trials?
4. What is the length of the epochs?
5. It is stated that the variance of EEG relative power was calculated by taking the standard deviation of the peak power of the entire frequency band (0 – 30 Hz) across the channels. I wonder if it would make sense to use the variance of EEG power for a certain frequency band (e.g., theta, alpha, beta) to control for the potential effect of artifacts (e.g., movements) on the high frequency bins. Given the PSD distribution for infants at this age, is it likely that the peak power will always reside in the low frequency bins in the theta band if there is no artificial effect on the higher frequency bins? 
6. What is the attrition rate of the current EEG analysis?
7. Under the section of “prediction of AR Status”, machine learning results were reported. They should belong to the results section. 
8. It seems to me that the authors tested a few machine learning algorithms using the EEG raw and relative power as predictors. Selection among these machine learning algorithms with the full dataset can render circular an otherwise appropriate analysis and “the best” results, which is called “circular analysis” or “double dipping” – the use of the same data for selection and data analysis. There are a few issues associated with circular analysis (Kriegeskirte et al., 2009) and neuroscientists tend to avoid spurious effects related to double dipping by using separate datasets for model selection and testing. In your situation, if using EEG variance of power and machine learning approaches to predict cognitive outcomes is one of the ultimate goals, I would recommend you select a machine learning algorithm (e.g., SVM or K-nearest Neighbor) based on a portion of the dataset (e.g., a few subjects) and then only apply this algorithm to the rest (or all) of the data in your future research.
9. Why did the authors use a logistic regression for variance of power but machine learning approaches for raw and relative power?
10. There is typo at the beginning of the section “Prediction of same visit (1 ^st^visit) Bayley scores. “Multivariable linear regression”. Should it be “multivariate linear regression” or “multiple linear regression”? 
11. Page 5, “Corresponding null models to null models plus variance of relative power … Bayley scores.” This sentence is confusing to me. Please clarify.  
12. In table 2, do those ANOVA p-values represent the significance of the whole multivariate regression model, or the t-test p-values for one independent factor, i.e., variance of relative power? My understanding is the latter, but please clarify it. 
13. Many analyses were done with these data (N >= 24?). Is the .05 significance level appropriate? Should it be adjusted?
14. Does “at-risk status” also predict concurrent or future Bayley scores? It would be great to see the results and discussion for this factor. 
15. Why is variance of EEG power predictive of some concurrent subscales of Bayley (e.g., raw fine motor, raw cognitive score, and total raw score) but not the others? Why is it only predictive of future Bayley raw fine motor score? This would be an interesting point for further discussion. 
16. This is a perplexing situation. On Page 8, I agree with the authors’ statement that individual EEG predictors may have limited power in predicting cognitive outcomes for infants and children. Then why not use a combination of EEG measures (e.g., power, variance, coherence, synchronization of oscillations, etc.) to predict the outcomes and infants’ developmental status, as some of these variables are already available to the authors? Will aggregate these measures in one model result in the highest prediction accuracy? Does any of the measures outperform the other measures? I feel these are important questions relevant to the current study and should be tested or discussed.

I have read this submission. I believe that I have an appropriate level of expertise to confirm that it is of an acceptable scientific standard, however I have significant reservations, as outlined above.

# Response

**Published:** 2018-11-07

**Authors:** Beth A. Smith

Thank you for your insightful review. In response: 

1. We appreciate this point and have been careful to say that higher variance in EEG power may represent less organized cortical activity, as opposed to state that it does represent less organized cortical activity. We have added the possibility of noisier data due to extensive eye movements for some infant participants in the limitations section. With such factors in mind, we carefully designed the experiment, e.g., introducing a spinning globe, to attract subjects’ attention, and rejected eye movement artifacts during preprocessing steps using independent component analysis to minimize the effect from EEG artifacts. We also added a sentence in the limitations section,  “It is also important to note that EEG is not a direct measure of cortical activity, so our proposal that higher variance may represent less organized cortical activity may or may not be valid. Future work that directly measures cortical activity is needed.”

2. This is an exploratory study to shed light on new potential metrics in EEG that could serve as features to capture early risk stratification and outcome prediction for infants at risk of developmental disability. Out of the available data, that being power and relative power of each electrode, variance of relative power was the measure that worked best.

3. The reviewer is correct regarding the study design. Only resting state data were analyzed here. We have added a sentence in the data analyses section, Only resting state EEG data were analyzed here, ranging from 14-82 seconds”.

4. We have added a sentence in the data analyses section, Only resting state EEG data were analyzed here, ranging from 14-82 seconds”.

5. We have looked at power variance in the individual frequency bins and peak power is typically within theta. One analysis looking at the alpha frequency was also predictive of 3rd visit scores using 1st visit resting state EEG. We did try prediction within each frequency brand specifically and we found looking across the spectrum to be the most robust.

6. A total of 22 infants with TD and 11 infants AR participated. After rejection, remaining EEG data were: AR visit 1 = 11, AR visit 3 = 9, TD visit 1 = 21, TD visit 3 = 13. We have added information to a sentence in the “Data analyses” section so that it now reads, “After rejection, remaining EEG data from 11 infants AR and 22 infants with TD were: AR visit 1 = 11, AR visit 3 = 9, TD visit 1 = 21, TD visit 3 = 13.”

7. We deleted the text “and only modest accuracy was identified with typically a high false negative rate” from the methods section and added the sentence “Leave-one-out cross-validation was performed on each machine learning model to predict at-risk status among 32 infants (11 at-risk) with a mean age of 90 days. Only modest accuracy was identified with typically a high false negative rate for features from conventional metrics (i.e., power and relative power)” to the results section.

8. We understand your concern, but we do not feel the initial use of machine learning on the entire data set would be considered double dipping. This is an exploratory study and the data set here was generated for the purposes of selecting a machine learning/statistical model. Future data collections of this work would use the same data analysis to further confirm and add to the existing data set as an independent set. We are really only advocating that variance of power is a variable of interest to be pursued.

9. We tested various machine learning algorithms including logistic regression, one of the simplest linear models, using conventional EEG metrics (power and relative power) as input features for the AR classification task. We have updated the description in our methods and results to clarify this. All models yielded poor performance with high false negative rate for these EEG metrics.  On the other hand, with the proposed variance of relative power, the simple logistic regression already demonstrated improved accuracy over the other EEG metrics, for the classification task.

10. Thank you for catching this, we have corrected it as “multivariate linear regression”.

11. We have clarified this statement as follows: “A baseline statistical model (a model that only included age in days and at-risk status) was compared to a nested model of the baseline model features plus variance of relative power to determine significant predictive effects of variance of relative power beyond baseline prediction. We used analysis of variance to determine significant predictive effects of variance of relative power across Bayley scores.”

12. The ANOVA p-values represent the probability that the more complex multivariable linear regression model, baseline plus variance of relative power, is significantly better than the baseline model alone. Specifically, the p-value is from the computation of an F-test between the two models to determine if the sum of squares for the more complex model is significantly different than the simpler model. This is only appropriate when the complex model is a nested version of the simple model, which is what we have done here.

13. No. The many analyses here were each is done independently of one another. One test for each outcome variable, one dependent variable to each independent variable. A case for alpha value adjustment, such as a Bonferroni correction, to ensure an adequate false positive rate, would be in the case of multiple comparison where it is multiple independent variables for one dependent variable. For example, multiple t-tests to determine if several independent variables are significantly different than the dependent variable.

14. At-risk status does not predict concurrent Bayley scores but it does predict future (3rd visit) Bayley score of raw receptive language, p < .05, R^2 = .18.

15. We agree with the Reviewer that this is a very interesting consideration. Perhaps movement requires a more *stable *brain than other measures? Or the movement measures are more sensitive of brain behavior than the other tests? We are really not comfortable speculating on this in the manuscript, though, as this is such an exploratory study.

16. We hope to explore this in future work. We have added in the Limitations and Future Directions section, “We hope to pursue larger, more complex predictive models in future work with a larger sample. Adding EEG measures such as coherence and synchronization of oscillations might increase predictive power, so might including structural brain imaging data or clinic variables.”
